# Behavioral factors and SARS-CoV-2 transmission heterogeneity within a household cohort in Costa Rica

**DOI:** 10.21203/rs.3.rs-2065331/v1

**Published:** 2022-09-19

**Authors:** Kaiyuan Sun, Viviana Loria, Amada Aparicio, Carolina Porras, Juan Carlos Vanegas, Michael Zúñiga, Melvin Morera, Carlos Avila, Arturo Abdelnour, Mitch Gail, Ruth Pfeiffer, Jeffrey Cohen, Peter Burbelo, Mehdi Abed, Cecile Viboud, Allan Hildesheim, Rolando Herrero, D Rebecca Prevots

## Abstract

Variability in household secondary attack rates (SAR) and transmission risks factors of SARS-CoV-2 remain poorly understood. To characterize SARS-CoV-2 transmission in a household setting, we conducted a household serologic study of SARS-CoV-2 in Costa Rica, with SARS-CoV-2 index cases selected from a larger prospective cohort study and their household contacts were enrolled. A total of 719 household contacts of 304 household index cases were enrolled from November 21, 2020, through July 31, 2021. Demographic, clinical, and behavioral information was collected from the index cases and their household contacts. Blood specimens were collected from contacts within 30-60 days of index case diagnosis; and serum was tested for presence of spike and nucleocapsid SARS-CoV-2 IgG antibodies. Evidence of SARS-CoV-2 prior infections among household contacts was defined based on the presence of both spike and nucleocapsid antibodies. To avoid making strong assumptions that the index case was the sole source of infections among household contacts, we fitted a chain binomial model to the serologic data, which allowed us to account for exogenous community infection risk as well as potential multi-generational transmissions within the household. Overall seroprevalence was 53% (95% confidence interval (CI) 48% – 58%) among household contacts The estimated household secondary attack rate (SAR) was 32% (95% CI 5% – 74%) and the average community infection risk was 19% (95% CI 14% - 26%). Mask wearing by the index case was associated with the household transmission risk reduction by 67% (adjusted odds ratio = 0.33 with 95% CI: 0.09-0.75) and sleeping in a separate bedroom from the index case reduced the risk of household transmission by 78% (adjusted odds ratio = 0.22 with 95% CI 0.10-0.41). The estimated distribution of household secondary attack rates was highly heterogeneous across index cases, with 30% of index cases being the source for 80% of secondary cases. Modeling analysis suggests behavioral factors were important drivers of the observed SARS-CoV-2 transmission heterogeneity within the household.

## Introduction

The household has been recognized as one of the main settings for SARS-CoV-2 transmission[1] with high secondary attack rates reported among household contacts[2] across multiple countries and in different phases of the pandemic[3–6]. Even after the initial acute phase of the pandemic, public health agencies in many countries recommended home-based isolation for people with confirmed SARS-CoV-2 infections to reduce overall community transmission[7]. However, for vulnerable individuals, having a household contact with confirmed SARS-CoV-2 infection greatly increases the risk of infection, which could lead to hospitalization or even death. While vaccination became available in 2021 in many countries with high effectiveness against symptomatic infections, the emergence of high-transmissibility and immune-escape variants, such as Omicron, along with waning immunity, have rekindled the importance of non-pharmaceutical interventions. Public health agencies have provided guidelines to reduce transmission within a household setting, including mask wearing and living in separate bedrooms[7], the effectiveness of such guidelines remain largely untested with real-world data.

Several household transmission studies have been conducted in high income countries, however, data from low- and middle-income countries are limited. Costa Rica has a universal health care system with a good infrastructure and robust surveillance system, which is ideal for conducting population-based transmission studies. Health care is centralized under the Costa Rican Social Security (Caja Costaricense de Seguro Social- CCSS) and most patients with COVID-19 are treated and followed at one of its health facilities with detailed records kept. The first case of COVID-19 in Costa Rica was detected on March 6, 2020, and soon after the CCSS Ministry of Health implemented population-level intervention measures including school closings and isolation at home for positive cases[8].

To better estimate the secondary attack rates and understand the behavioral determinants of SARS-CoV-2 household transmission, we conducted a household serologic study nested within a larger prospective population-based study of the SARS-CoV-2 immunologic response in Costa Rica. We fit the serologic data to a chain-binomial household transmission model to account for the non-linear transmission dynamics as well as the time-varying community infection risk. Moreover, this model is able to incorporate detailed demographic, clinical and behavior risk factors of the index and household contacts. We estimated the overall household secondary attack rate, the cumulative community infection risk, and assessed sources of transmission heterogeneities among household members.

## Results

### Study overview

From December 1, 2020, through July 30, 2021, a total of 986 household contacts were approached of whom 719 (73%) consented to enroll in the household study. These contacts were distributed in 304 households. This study period covered the first wave and the beginning of the second wave in Costa Rica (S1). The total household size ranged from 2 to 9 with an average household size of 3.3. Among 304 index cases, the median age was 38 (range: 0 – 101); 163 (54%) index cases were female. Among 719 household contacts, the median age was 32 (range: 0 – 93); 404 (56%) household contacts were female. Less than 10% of the study participants were vaccinated with at least one vaccine dose. Detailed demographic, clinical, and behavioral factors for both the index case and the household contacts are summarized in Table 1.

### Seroprevalence among household contacts

To evaluate the burden of SARS-CoV-2 among 719 household contacts, we estimated the seroprevalence both overall and within strata, defined by: household size; age, sex, and obesity status of the index case and household contacts; and behavioral factors such as mask usage and interactions of the contact with the index case (Figure 1). The seroprevalence along with 95% CI were estimated using univariate generalized estimating equations with household clustering. The overall seroprevalence was 53% (95% CI 48% – 58%) among household contacts, but seroprevalence varied substantially across different strata. In particular, for behavioral risk factors, the seroprevalence was higher if the contact cared for the index case (59% with 95% CI 53% - 65% vs 48% with 95% CI 44% - 55%, $p>0.05$), shared a bedroom with the index case (67% with 95% CI 59% - 73%) vs 48% ( 95% CI 43% - 54%, $p<0.001$) or had interactions with the index case outside the bedroom (58% with 95% CI 53% - 63% for >1 hours vs 41% with 95% CI 34% - 50% for <1 hour, trend test $p<0.001$).

Age-specific mixing patterns between the index case and household contacts are shown in Figure S2A. The household mixing patterns in Costa Rica resemble those observed in other countries[9,10], with a distinct "three-band" feature. The diagonal band represents mixing with contacts of approximately the same age, while the two off-diagonal bands representing inter-generational mixing (parents living with young kids/adults living with elder parents). The mixing pattern between index and seropositive household contacts is distinctly different from that between index and seronegative contacts (Figure S2 B-C), suggesting age may be a significant risk factor associated with SARS-CoV-2 transmission. We thus further explored age as a risk factor by including age variables modulating infectivity and susceptibility in the chain-binomial household transmission models.

### Fitting chain-binomial household transmission model to the SARS-CoV-2 serologic data

The chain-binomial household transmission model fitted to serologic data revealed multiple risk factors associated with household transmission of SARS-CoV-2. We found that incorporating cumulative incidence rate in Costa Rica as a coefficient for community infection risk improved the fit of the model (Table S1 Model 1 vs Model 0), suggesting community infection risk correlates with SARS-CoV-2 circulation intensity outside the household. We found that asymptomatic index individuals were as likely to transmit SARS-CoV-2 as symptomatic index cases, confirming the significant contribution of asymptomatic transmission in the spreading of SARS-CoV-2 (Figure 2A)[11]. Importantly, we found that behavioral factors were significant drivers of household transmission: sharing a bedroom with the index case (adjusted odds ratio not sharing vs. sharing: 0.22 with 95% CI (0.10 – 0.41), or caring for the index case (adjusted odds ratio not caring vs caring: 0.45 with 95% CI 0.19 – 0.89) were risk factors for transmission, while the index case wearing a mask (more than half of the time during the two weeks post diagnosis, adjusted odds ratio 0.33 with 95% CI 0.09 – 0.75) was protective. Avoiding interaction with the index case (<1 hour) within two weeks of his/her diagnosis would reduce the risk by 45% (adjusted odds ratio vs >1 hours: 0.55 with 95%CI: 0.34 – 0.86 ). Interestingly, whether household members wore a mask or not when interacting with the index case did not significantly affect the risk of acquiring infection. Our model suggests that the number of household contacts had a strong negative association with the per-contact risk of SARS-CoV-2 transmission: doubling the number of contact numbers decreases the per-contact risk of transmission by 74% (95%CI: 67% - 79%). In addition, gender was neither significantly associated with SARS-CoV-2 susceptibility nor infectivity. We did not observe a significant association between age of the index case and SARS-CoV-2 infectivity but found a significant association between age of the household member and SARS-CoV-2 susceptibility: children under the age of 12 were significantly more likely to be infected when compared to age group 40-59 (OR 1.57, 95%CI: 1.08-2.28), while all other age groups were significantly less susceptible (Figure 2A).

Utilizing the best-fitting estimate of the chain-binomial model, we projected the distribution of the community infection risk as well as the household secondary attack rate across all cohort participants. We estimated that the average cumulative community infection risk during the study period was at 19% (95%CI: 14%-26%, Figure 2C), lower than the household secondary attack rate attributable to seropositive household members (32%; 95%CI 5%-74%, Figure 2D). Interestingly, the average projected secondary attack rate by the index case was 12% (95%CI 0%-63%), less than half of the secondary attack rate attributable to seropositive household members (Figure 2E). This finding is explained by the fact that a significant fraction of the cohort population took protective measures after diagnosis of the index case, shown to be effective at reducing transmission, including avoiding sharing a bedroom, reducing interactions outside the bedroom, and wearing masks (Table 1). We also found that 30% of index cases were the source for 80% of all secondary cases' onward transmission, indicating high transmission heterogeneity (Figure 2E).

We further projected a hypothetical scenario in which the cohort population did not adopt preventive behavioral measures (all household members shared a bedroom with the index case, interacted with the index for more than 10 hours outside the bedroom, took care of the index case who did not wear a mask most of the time). The projected secondary attack rate by the index case was 37% (95%CI 5%-82%), comparable to the secondary attack rate attributable to seropositive household members (Figure 2F). In this case, transmission heterogeneity would be much reduced (Figure 2G), with 58% of the index cases being the source for 80% of onward transmission, suggesting variation in the adoption of preventive behavioral measures were major sources of index case's observed transmission heterogeneity. If, on the contrary, all behavioral risk factors were avoided and preventive measures adopted, the secondary attack rate by the index case could be reduced to 3% (95%CI: 0%-11%). We further conducted a sensitivity analysis of bootstrapping estimates at the households' level, controlling for the joint distribution of household size and age category (Table 1), sex, and diagnostic month of the index cases (detailed in , to address potential household clustering effect (Figure S4). The bootstrapping confidence intervals (Figure S4) are wider than likelihood-ratio based confidence intervals (Figure 2A), with the effects of index case mask wearing and duration of interaction outside the bedroom (with index case) become statistically non-significant.

### Symptoms associated with SARS-CoV-2 seropositivity

We surveyed the presence of fourteen COVID-19 related symptoms independent of serostatus. The prevalence of each symptom is presented in Figure 3A, along with the relative risk comparing seropositive to seronegative contacts in Figure 3B. Overall, the prevalence was significantly higher for all 14 symptoms for seropositive individuals than for those who were seronegative (Relative Risk (RR]>2, p<0.01 for all 14 symptoms). For symptoms with a higher than 20% prevalence among seropositive individuals, loss of smell (RR=5.5, 95% CI 5.0 – 6.0) and loss of taste (RR=4.7, 95% CI 4.4 – 5.0) were the most predictive of SARS-CoV-2 infection. Seventy percent of seropositive individuals had at least one symptom, while only 29% of seronegative individuals reported at least one symptom (Figure 3C). Logistic regression of having at least one symptom against an indicator of seropositivity yielded an adjusted odds ratio of 9.2 (95% CI 4.6 – 18.5, p<0.001). However, among seropositive individuals, the prevalence of symptom presentation differed significantly by age: persons aged 0-12 and 13-24 years were 72% and 69% less likely to be symptomatic (OR 0.28 with 95% CI 0.1 – 0.77 and 0.31 with 95% CI 0.11 – 0.85 respectively, p<0.05 for both) compared with persons aged 40-59 years.

## Discussion

Through fitting transmission models to household serologic data, we obtained estimates for both secondary attack rates and community transmission and identified significant behavioral measures for preventing household transmission in the pre-vaccination and pre-immune escape variant era. Although seroprevalence and household studies have been conducted in Latin America[12,13] our study is the first to estimate both household secondary attack rate and community infection rates, and to identify specific actionable preventive measures. This work adds to our knowledge of SARS-CoV-2 transmission in middle-income countries in Latin America, and more broadly expands our understanding of transmission in a variety of settings.

A highlight of our study is that it provides real-world evidence that preventive measures within the household, such as sleeping arrangements and reducing contacts outside the bedroom, as well as household members and infected individuals wearing masks, could significantly reduce the risk of SARS-CoV-2 transmission within the household. Interestingly, we found that masks wearing by the index case is effective as "source control". A recent household study conducted during the Omicron wave in four jurisdictions in the United States similarly found that attack rates were significantly lower among index cases who isolated or wore a mask[1]. In addition, a 2019 study of household transmission following a summer camp outbreak included 224 index cases ages 7-19 with 377 contacts tested; a strong protective although non-significant effect of index case masking was found. Our study emphasizes the importance of non-pharmaceutical interventions in reducing infection risk and disease burden in the household setting, especially when vaccines are not widely available or ineffective in preventing transmission.

We found that children aged <12 years were more likely to become infected. Age as a risk factor for susceptibility and transmissibility has been studied in numerous settings and a variety of designs; the effect of age is highly dependent on age-specific contact rates and is therefore difficult to disentangle from biologic effects[14]. The increased susceptibility of the <12 age group may be a function of behavioral factors, particularly time spent at home, as children in this age group are more likely to remain home under adult supervision and therefore have a higher risk of exposure to and in-home transmission from adult contacts. We also found that obesity significantly increased susceptibility to SARS-CoV-2. Similar associations have also been observed in a household cohort in South Africa as well as for other respiratory viruses such as influenza A H1N1pdm[15,16].

Prior studies have shown that children tend to have milder infections, and are more likely to have upper respiratory infections relative to adults[17], which is confirmed in our study. Our study is however unique in that both seropositive and seronegative individuals were asked about their symptom presentation around the time of the diagnosis of the index case, prior to the serum sample collection. The seronegative individuals served as a control group to assess symptom prevalence in non-infected populations as many of the COVID-19 related symptoms are nonspecific. Our results confirm the high rate of asymptomatic infections in the younger population and identified loss-of-taste and loss-of-smell as highly specific to SARS-CoV-2 in the pre-Omicron era.

To further explore the importance of household size and contacts, we tried models assuming logarithmic and linear relationships for the number of households contacts and found that the logarithmic model performed best (Table S1, Model 7 vs. Model 8). This suggests a power law relationship between household secondary attack rate and the number of household contacts $n$, i.e.: $SAR \propto n^{-\gamma}$ where $\gamma = 1.7$ was estimated by our model. We found that the household secondary attack rate decreased when household size increased and that a power law relationship linked household secondary attack rate with the number of household contacts $n$, where

$$SAR \propto n^{-\gamma}$$

and $\gamma = 1.7$. This could be due to a dilution of household interaction intensity per household contact, whereby an individual in a large household has more household members for interactions than in a small household, and hence less propensity to interact with the index case.

The chain-binomial model revealed that the distribution of secondary attack rate by the index case is highly heterogeneous, with 30% of index cases being the source for 80% of all secondary cases' onward transmission (Figure 2E). This heterogeneity was mainly driven by the partial adoption of the preventive measures. In the hypothetical scenario without any preventive measures (Figure 2F), the transmission heterogeneity would be much reduced, with 6% of the index cases being the source for 80% of onward transmission. This suggests that variations in the adoption of preventive measures contribute to the observed heterogeneities in SARS-CoV-2 transmission chains[2,18]. Comparison of secondary attack rates across studies is limited by differences in study design, including infection ascertainment as well as follow-up and approaches for SARS-CoV-2 antigen or antibody testing. However, our secondary attack rate is somewhat higher than the SAR of 23.9% found in a large household cohort observed in South Africa from July 2020 -August 2021[4], and is lower than that found in household studies in the United States, with SARs of 61% for Alpha variants and 55% for non-Alpha variants[3]. A household-based community cohort study conducted in Nicaragua during March 2020-2021 found a seroprevalence of 57% after the first epidemic wave, comparable to our cumulative infection rate of 53%[12]; however, SARs were not reported.

Our study has several limitations. First, questionnaires related to behavioral factors (sharing a bedroom, interaction outside the bedroom, and caring for the index case) were only directed towards the interaction between the index individual and each household member. We could not evaluate how the interactions between (non-index) household members impact transmission. Second, we could not assess how variations in viral shedding duration and intensity across infected individuals could potentially affect transmission, as we did not collect respiratory samples from the participants. In particular, a recent study from South Africa has shown the importance of viral load and kinetics on SARS-CoV-2 household transmission[19]. We also did not assess household ventilation parameters which could impact SARS-CoV-2 transmission risk within confined space[20]. Finally, these estimates were from the first wave, and may not be generalizable to later epidemic waves with more transmissible or immune escape variants. However, these estimates serve as a baseline for future studies, and our findings regarding household prevention here are comparable to those found in the U.S. during the Omicron wave, suggesting the generalizability of the findings. In summary, our study from a middle-income country in Latin America points to relatively simple preventive measures to limit household transmission and suggests that simple behavioral mechanisms can explain the pervasive transmission heterogeneity reported in SARS-CoV-2.

## Methods

**Study population**

For the larger prospective study (RESPIRA), 1000 cases were recruited from three geographic areas: Puntarenas Province, Greater San Jose Metropolitan Area - (Gran Area Metropolitano), and the province of Guanacaste, and four age strata (0-19, 20-39, 40-59, 60+) using national surveillance lists provided by the CCSS and Health Ministry. The geographic areas were selected based on logistic considerations and represented 58% percent of the Costa Rican population. Cases were sampled randomly within each geographic area and age stratum. . Approximately 30% of cases were approached for consent to participate in the nested household study; these cases were termed “index” cases.

A household was defined as two or more people living together who shared a kitchen. To be eligible for inclusion, a contact must have spent at least one night per week in the living area since the diagnosis of the index case. After consent and enrollment, index cases and their household contacts were administered a questionnaire to ascertain demographic, clinical, and behavioral risk and preventive factors. For household contacts, symptoms related to SARS-CoV-2 were ascertained for the time period two weeks before or two weeks after the sample collection date for the index case (referred to hereafter as “date of diagnosis”). If a household contact reported a prior diagnosis of COVID, symptoms were ascertained in relation to that diagnosis. Blood samples were collected from household contacts 30 to 60 days after the date of collection of the PCR-confirmed positive sample of the index case, and serum samples were tested to ascertain the presence of SARS-CoV-2 antibodies (against both SARS-CoV-2 nucleocapsid and spike protein), as a marker of past SARS-CoV-2 infection.

Household index cases and their contacts were enrolled from December 1, 2020, through July 31, 2021. This period coincided with the middle of the first wave and the end of the second wave in Costa Rica (Figure S1). The study was conducted immediately prior to the widespread availability of SARS-CoV-2 vaccines in Costa Rica.

The RESPIRA study protocol was approved by the Central Institutional Review Board of the CCSS. (protocol R020-SABI-000261). Informed, signed consent was obtained from all study participants or their proxies.

**Serologic Methods**

Serum samples were tested for the presence of SARS-CoV-2 spike and nucleocapsid anti-IgG antibodies using a previously validated quantitative immunoprecipitation assay in a microtiter plate format[21]. We defined seropositivity as positive to both spike and nucleocapsid antigens and considered it evidence of past SARS-CoV-2 infection. The serum samples were collected between 30 and 60 days after the index case PCR positive sample collection to allow time for seroconversion. Approximately 7.5% of the samples were incorporated into the plates in a blinded fashion to evaluate within and between plate variability. The one-way Intraclass correlation coefficient (ICC) for nucleocapsid within-plate duplicate was 0.94 with 95% CI 0.87 – 0.97; the ICC for spike within-plate duplicate was 0.95 with 95% CI 0.89 – 0.98; the ICC for nucleocapsid across-plate duplicate was 0.71 with 95% CI 0.44 – 0.87; the ICC for spike within-plate duplicate was 0.87 with 95% CI 0.72 – 0.94. In addition 25 pre-pandemic samples from a population study in Costa Rica[22] were tested as negative controls to ensure assay validity; all were classified as seronegative, as expected.

**Chain binomial household transmission model**

Here we consider a multi-variable chain-binomial household transmission model for SARS-CoV-2, as an extension of prior household models developed to study influenza transmission[23,24]. The model was fitted to the cumulative outbreak size at the end of the household outbreak, i.e., the total number of people infected, rather than the precise sequence and timeline of infections. We do not assume that all seropositive household members acquired infections from the index case and allow for community-acquired infections (prior to blood sample collection) for household members and multigenerational transmission within the household.

Specifically, let $h$ denote a household, $i$ an individual, with $i_-^h$ an individual $i$ who is serologic negative in household $h$ and $i_+^h$ an individual $i$ who is serologic positive in household $h$. The risk of acquiring infection from the community varies over time due to changing incidence and is written as $P_c * f(t)$ , where $f(t)$ is the cumulative incidence rate from the start of the pandemic until time $t$ in Costa Rica, and $P_c$ is the baseline community infection risk to be estimated by the model. If we denote $t_s^i$ the time of serology sample collection for household member $i$, then the likelihood of an individual $i$ escaping infection from the community is given by:

$$l_c^i = 1 - P_c * f(t_s^i)$$

To model the risk of transmission between the index case and household members, we denote $P_h^{ic-j}$ as the risk of index case $ic$ infecting household member $j$ in household $h$. We can express $P_h^{ic-j}$ as:

$$P_h^{ic-j} = P_{index} \exp\left(\sum_k \alpha_k a_k\right)$$

where $P_{index}$ denotes the baseline risk of SARS-CoV-2 transmission within the household and $a_k$ represents risk factor $k$ that could potentially influence household transmission risk. We include risk factors affecting transmissibility and susceptibility such as age, sex, and obesity status (self-reported) of the index case $ic$ and of household contacts $j$. We also include household size, and behavioral factors such as whether the contact shared a bedroom with the index case, spent time with the index case outside the bedroom, cared for the index case, and wore a mask following diagnosis in the index case (index case and household member). We examine a sequence of models including various risk factors as shown in Supplemental Table S1. We can then express the likelihood of household member $i$ escaping infection from the index case as:

$$l_{index}^{i} = \left(1 - P_h^{ic-j}\right)$$

To model the risk of transmission between household contacts (in i.e., chains of transmission that do not involve the index case), we denote $P_h^{ij}$ as the risk of seropositive household contact $i$ infecting household contact $j$ in household $h$. We can express $P_h^{ij}$ as:

$$P_h^{ij} = P_{hh} \exp\left(\sum_k \beta_k b_k\right)$$

where $P_{hh}$ denotes the baseline risk of SARS-CoV-2 transmission between an infected household contact and another uninfected household contact and $b_k$ represents risk factor $k$ that could potentially influence household transmission risk. We consider a different baseline transmission risk from an average household member ($P_{hh}$) vs the index case ($P_{index}$) because we have slightly different behavioral variables collected from household members and index cases, especially regarding all the pairwise interactions between index case and household members. This does not assume an inherently different biologic risk from index cases and household members, but merely measurement differences. Here we include risk factors including age, sex, and obesity status of the seropositive infector $i$ (modulating transmissibility) as well as household contacts $j$ (modulating susceptibility). We also include household size, whether the recipient wears a mask, and the symptom status of the infector.

We can then express the probability of household contact $i$ escaping infection from all positive household contacts as:

$$l_{hh}^{i} = \prod_{\{j \neq i\}} \left(1 - P_h^{ij}\right)$$

where $\{j \neq i\}$ represents all positive household contacts. Thus, within household $h$, the likelihood of household contact $i$ being seronegative at the end of the household outbreak is given by:

$$l_{-}^{i} = l_c^{i} l_{index}^{i} l_{hh}^{i}$$

And the likelihood of an individual $i$ being seropositive is given by:

$$l_{+}^{i} = 1 - l_{c}^{i} l_{index}^{i} l_{hh}^{i}$$

For household $h$, the loglikelihood of observing the infection status of all household contacts is given by:

$$\log(l^{h}) = \sum_{\{i_{+}^{h}\}} \log(l_{+}^{i}) + \sum_{\{i_{-}^{h}\}} \log(l_{-}^{i})$$

The overall likelihood of the observations across all households is given by:

$$\log(L) = \sum_{h} \log(l^{h})$$

We fit the model to serology observations and used maximum likelihood method to infer parameters: estimates on $P_c, P_{hh}, P_{index}$ reported in Figure 2B while $\{\alpha_k\}$ and $\{\beta_k\}$ estimates were reported in Figure 2A. 95% confidence intervals were determined by likelihood ratio test. To address potential household clustering effect, we bootstrapped over 304 households, controlling for the household size distribution as well as the distribution of age category, sex and diagnostic month of the index cases (i.e., for each bootstrap samples, the joint distribution of household size, index case's age category (see Table 1 for the age strata), index case's sex, and index case's diagnostic month is the same as the original data. We use 100 times repeated bootstrapped estimates to construct bootstrapping confidence intervals as a sensitivity analysis.

## Data Availability

The investigators welcome inquiries about possible collaborations and requests for access to the dataset. Anonymized datasets as required can be shared after approval of a proposal and a signed data use agreement. Investigators interested in more details about this study, including protocols and informed consent forms should contact the principal investigator and corresponding author, D Rebecca Prevots (rprevots@niaid.nih.gov).

## Author contributions.

DRP: Study conception, study design, data analysis, manuscript writing.

KS: data analysis, manuscript writing, verified the underlying data.

VL, AA, CP, JCV, MZ, MM, CA, AA, AH, RH - study design, data acquisition, data analysis, manuscript revision

VL, JCV- verified underlying data.

MHG, RP, CV- study design, data analysis, manuscript revision.

JIC, PDB, MAA- serologic testing, data analysis, manuscript revision.

## Acknowledgements

This work was supported by the Intramural Research Programs of NIAID, NIDCR, NCI, and by the FIC. We wish to thank all participants in this study. We also thank Dr Aimee Kreimer for her support in implementing this study.

## Disclaimer

The findings and conclusions in this report are those of the authors and do not necessarily represent the official position of the NIH.

## Tables and Figures

**Table 1.** Characteristics of the studied population stratified by index case and household members.

| Characteristics | Index cases | Household contacts |
|---|---|---|
| **Age** | | |
| 0-12 years | 30/304 (10%) | 148/719 (21%) |
| 13-24 years | 53/304 (17%) | 125/719 (17%) |
| 25-39 years | 75/304 (25%) | 163/719 (23%) |
| 40-59 years | 80/304 (26%) | 171/719 (24%) |
| ≥ 60 years | 66/304 (22%) | 112/719 (16%) |
| **Sex** | | |
| Male | 141/304 (46%) | 315/719 (44%) |
| Female | 163/304 (54%) | 404/719 (56%) |
| **Obesity** | | |
| Yes | 36/304 (12%) | 41/719 ( 6%) |
| No | 268/304 (88%) | 678/719 (94%) |
| **Care for index case** | | |
| Yes | - | 274/719 (38%) |
| No | - | 359/719 (50%) |
| NA | - | 86/719 (12%) |
| **Sharing bedroom with index case (2 weeks post index diagnosis)** | | |
| Yes | - | 191/719 (27%) |
| No | - | 525/719 (73%) |
| NA | | 3/719 ( 3%) |
| **Hours (per day) with index case outside bedroom (2 weeks post index diagnosis)** | | |
| <1 hour | - | 236/719 (33%) |
| >1 hours | - | 473/719 (66%) |
| NA | - | 10/719 ( 1%) |
| **Index case mask wearing frequency* (2 weeks post index diagnosis)** | | |
| > half of the time | - | 189/719 (26%) |
| ≤ half of the time | - | 322/719 (45%) |
| NA | - | 208/719 (29%) |
| **Household contact mask wearing frequency* (2 weeks post index diagnosis)** | | |
| > half of the time | - | 184/719 (25%) |
| ≤ half of the time | - | 349/719 (49%) |
| NA | - | 186/719 (26%) |
| **Vaccination status** | | |
| Unvaccinated | 296/304 (97%) | 649/719 (90%) |
| 1st dose | 5/304 (2%) | 29/719 (4%) |
| 2nd dose | 3/304 (1%) | 41/719 (6%) |

*Here we looked at the mask wearing frequency of index case and household contact when they were interacting with each other under the household setting.

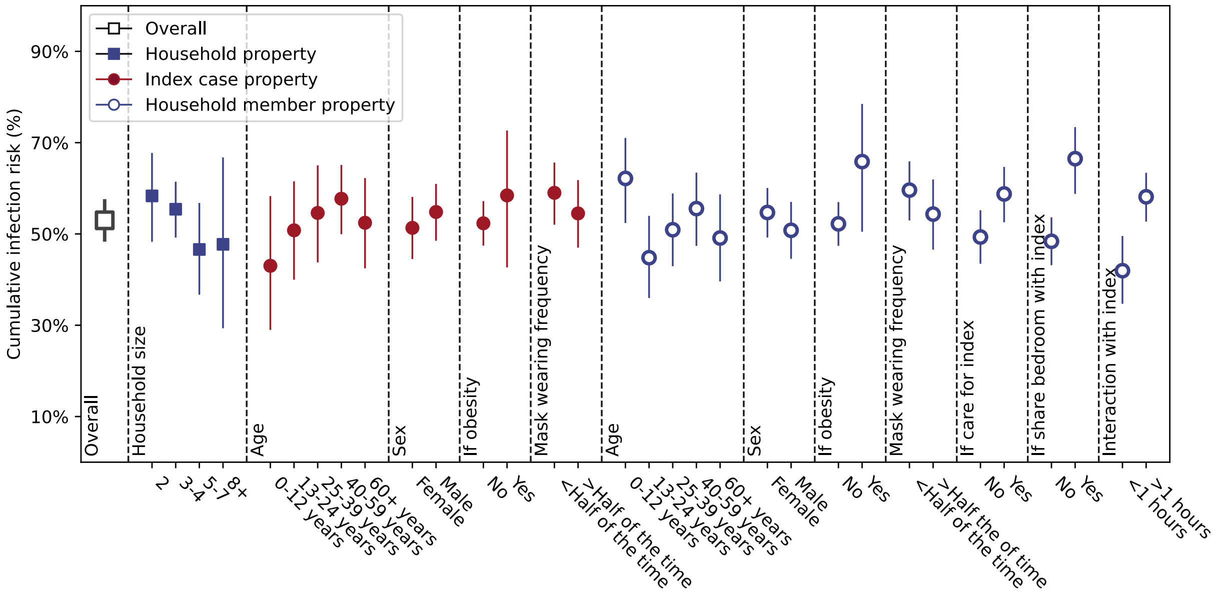

**Figure 1: The overall cumulative infection risk among household members and cumulative infection risk by different strata.** The overall cumulative infection risk is calculated as fraction of seropositive among all 719 household contacts. The cumulative infection risk within a given stratum is calculated as the fraction of seropositive individuals among the household contacts within the stratum. We stratified the 719 household contacts by household level property of household size; index case property including index cases' age, sex, obesity or not, mask wearing frequency; household member properties including household contacts' age, sex, obesity or not, mask wearing frequency, if cared for index case, shared bedroom with index case or interaction frequency with index case after the diagnosis of the index case. Confidence intervals are based on a generalized estimating equation analysis applied to each risk factor one at a time that takes within household correlations into account.

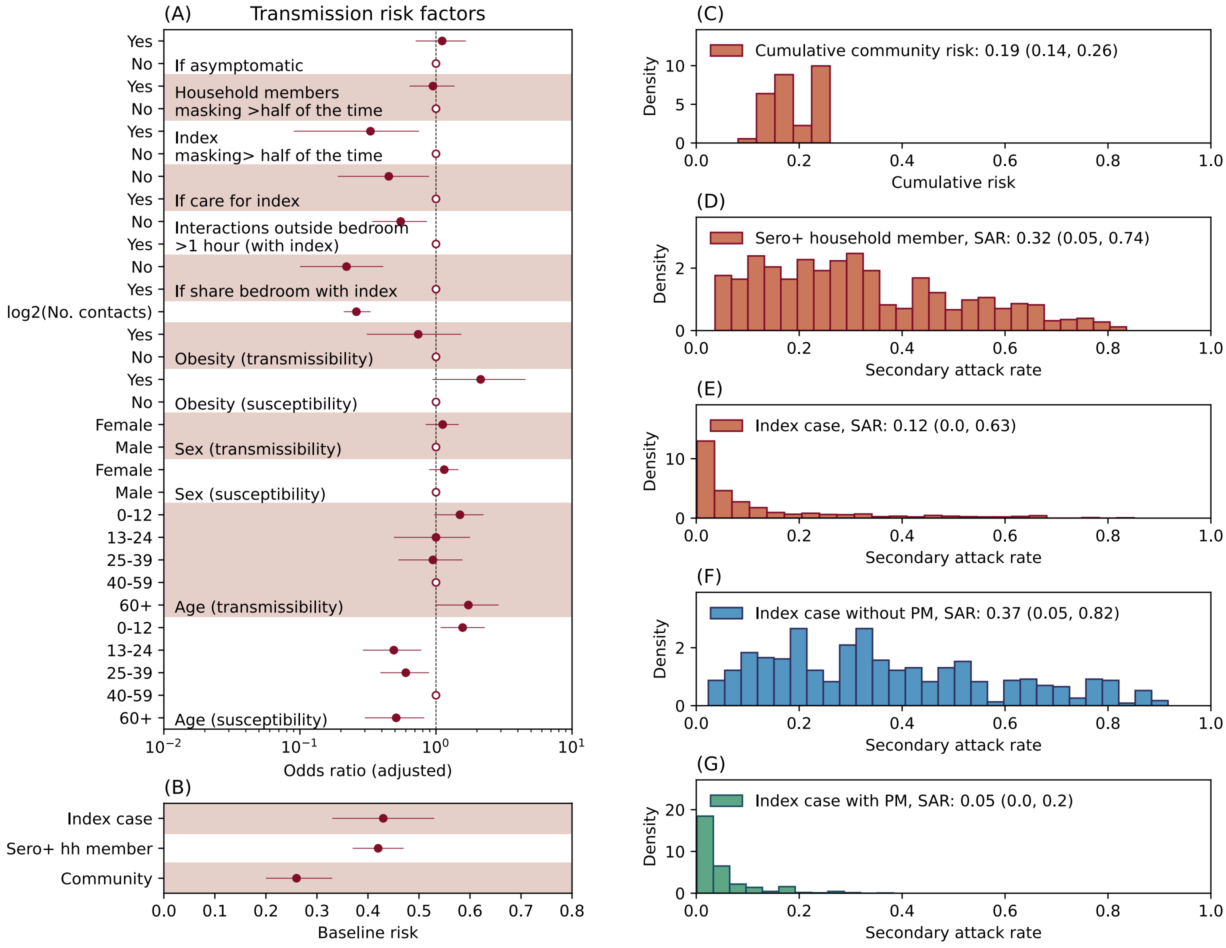

**Figure 2: Estimates from chain-binomial household transmission model.** (A) Estimated odds ratios (adjusted) of the transmission risk factors. Solid dots and horizontal lines represent point estimates and 95% confidence intervals. Circles represent the reference class. (B) Baseline transmission risks from the index case and seropositive household members as well as baseline risks of acquiring infection from the community. (C-F) Distribution (histogram) of model projected community infection risk and household secondary attack rate across the study participants. (C) Distribution of cumulative community infection risks* (D) Distribution of the secondary attack rate attributable to seropositive household members who are not the index cases (E) Distribution of the secondary attack rate attributable to the index case. (F) Distribution of the secondary attack rate by the index case in a counterfactual scenario where no preventive measures (PM) were taken after diagnosis of the index case. (F) Distribution of the secondary attack rate by the index case in a counterfactual scenario where all preventive measures (PM) were taken after diagnosis of the index case. (*All results are from model with best fit to the data: model 15, Table S1).

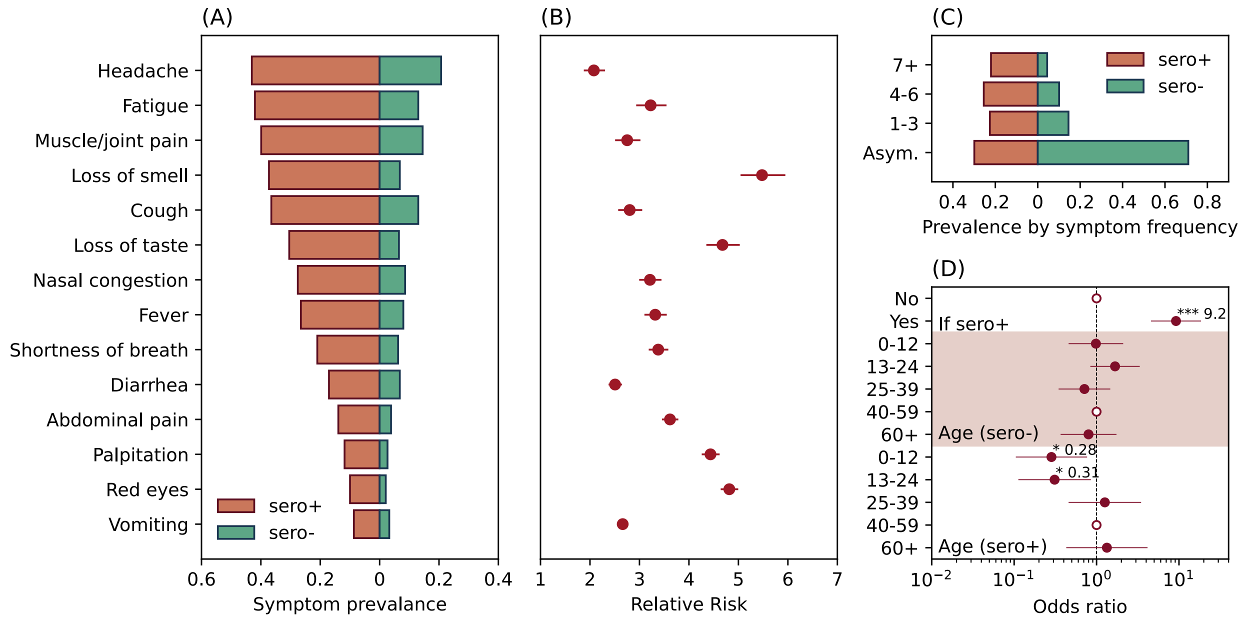

**Figure 3:** (A) Prevalence of symptoms among both seropositive (red bar) and seronegative individuals (green bar). (B) The relative risk of symptom presentation in seropositive vs seronegative individuals. Panel B share the same y axis as panel A. Dots and horizontal lines represent point estimate and 95% confidence interval. (C) The prevalence of symptoms by symptom frequency (Asym. denotes asymptomatic individuals, 1-3 denotes individuals having 1-3 of all symptoms listed in A, 4-6 denotes having 4-6 of all symptoms listed in A and 7+ denotes having more than 7 symptoms listed in A. (D) Regression analysis on the risk of being symptomatic (having a least 1 symptom in A by serologic status and age. “If sero+” denotes if the individual is seropositive; “Age (sero-)” denotes the age dependency of being symptomatic among seronegative individuals; Age (sero+) denotes the age dependency of being symptomatic among seropositive individuals

## Supplementary Files

This is a list of supplementary files associated with this preprint. Click to download.

- RESPIRAHHNatCommSI.pdf
